# Genetics of Thyroid-Stimulating Hormone Receptor—Relevance for Autoimmune Thyroid Disease

**DOI:** 10.3389/fendo.2017.00057

**Published:** 2017-04-03

**Authors:** Mihaela Stefan, Larissa C. Faustino

**Affiliations:** ^1^Division of Endocrinology, Department of Medicine, Albert Einstein College of Medicine, Bronx, NY, USA

**Keywords:** Graves’ disease, thyroid-stimulating hormone receptor, single nucleotide polymorphisms, GWAS, histone modifications

## Abstract

Production of thyroid-stimulating hormone receptor (TSHR) antibodies represents the hallmark of Graves’ disease (GD) pathogenesis. Thus, for more than two decades the TSHR gene has been at the center of studies intended to elucidate its contribution to disease pathology. The advent of genome-wide association technology allowed to establish a strong association of the TSHR gene with GD. Subsequent fine-mapping studies narrowed the disease-susceptibility region to a 40 kb sequence in intron 1, where at least five GD-associated SNPs in tight linkage disequilibrium were identified. The current challenge is to understand the functional mechanisms by which these polymorphisms modify physiological processes and trigger disease. The aim of this review is to summarize the current knowledge on the role of the TSHR gene in GD pathogenesis, which has been gained through linkage and association studies, as well as to discuss the emerging mechanisms underlying biological implications of TSHR variants in the development of GD.

## Introduction

Autoimmune thyroid diseases (AITD), including Graves’ disease (GD) and Hashimoto’s thyroiditis (HT), affect 2–5% of the general population, representing the most frequent autoimmune conditions (1, 2). Similar to other complex autoimmune diseases, it is believed that AITD occur when interactions of genetic susceptibility factors with environmental triggers lead to loss of immune self-tolerance at peripheral and central levels (3). During the last three decades, several approaches from linkage and association studies to candidate genes analysis and whole-genome screening enabled significant progress in the identification of genes that confer susceptibility to AITD. These genes are broadly grouped as either immune-modulating genes (including the HLA family, CD40, CD25, FOXP3, PTPN22) or thyroid-specific genes [thyroid-stimulating hormone receptor (TSHR) and TG].

The TSHR is unique among these susceptibility genes because it encodes for a protein that is both responsible for the clinical manifestations of the disease and is the direct target of autoimmune response in GD. The TSHR gene, located on chromosome 14q31, consists of 10 exons and encodes for a G protein-coupled receptor that plays a central role in the regulation of thyroid development, growth, and function. Indeed, TSHR-stimulating antibodies (TSAbs) are present in nearly all cases of GD, and severity of the disease correlates with TSAbs levels (4). Moreover, compelling evidence has correlated TSHR gene variants exclusively with GD susceptibility and not with HT development (5, 6). The aim of this review is to summarize the current knowledge of the role of TSHR gene in GD pathology, gained through linkage analysis, association, and functional studies.

## Linkage Studies

Whole-genome linkage is a powerful technique for screening the human genome for major susceptibility genes, without any previous assumption on the mechanisms of genetic susceptibility to the disease. It is based on the principle that the probability of recombination between two loci is directly related to the genetic distance between them. Thus, if two loci comprising a polymorphism and a disease-related gene are close to each other on a chromosome, the alleles of the polymorphic loci will cosegregate with the disease within affected families because the likelihood of recombination between the polymorphism and the disease-related gene is very low. Single nucleotide polymorphisms (SNPs) and microsatellites are the main genetic polymorphisms screened in the linkage studies (7, 8).

Several linkage studies intended to detect GD-specific loci had limited success in demonstrating significant linkage (as well as association) of the TSHR gene with GD (9–13). However, Tomer et al. identified a large region of linkage on chromosome 14q31 containing the TSHR locus in whole-genome linkage studies in multiplex, multigenerational AITD families (14–17). They identified a locus, designated Graves’ disease 1 (GD-1) that included TSHR gene, as well as other potential candidate genes—such as estrogen receptor 2, deiodinase type 2, and immunoglobulin heavy locus (14, 17). Later, fine-mapping analysis by Tomer et al. confirmed that the susceptibility gene in the GD-1 locus was indeed the TSHR gene, even though a second gene in this locus, NRXN3, was also identified as a major GD gene (18).

## Association Studies

Reflecting the importance of TSHR for GD pathogenesis, TSHR was the first non-MHC gene to be tested for association with the disease. Three germline missense mutations were initially described in patients with GD and proposed to be associated with the disease (19, 20): a substitution of aspartic acid (D) for histidine (H) in position 36 (D36H); a substitution of a proline (P) for threonine (T) in position 52 (P52T), and a substitution of aspartic acid (D) for glutamic acid (E) in position 727 (D727E). Two of these three mutations, D36H and P52T, are located in the putative ligand binding region of the extracellular domain of the TSHR, while the third one, D727E lies within the intracellular domain of the receptor.

However, studies regarding association of these SNPs with GD were contradictory. In 1995, Bahn and colleagues were first to report the association of the P52T polymorphism with AITD in female population (21), but in a subsequent study Watson et al. found no differences in the distribution of this polymorphism in GD patients as compared with autoimmune hypothyroidism patients and control individuals (22). Several subsequent studies reached inconsistent results, thus a clear association of P52T or D36H with GD was not confirmed (23–25). In 1999, Gabriel et al. reported that the C to G transition in the TSHR 727 codon leading to D727E variant has an increased frequency in patients with non-autoimmune thyroiditis (26), and this association was also recently reported in a small Turkish population (27). However, the D727E association with non-autoimmune thyroiditis was not supported by studies in large series of European Caucasian patients (28). The association of D727E polymorphism with autoimmune thyroiditis was supported by data from a case–control study in Russian populations (29) but was not confirmed in US Caucasian patients (13, 26). Finally, Tomer group performed a case–control study and meta-analysis combining the data from three independent studies and showed a very week association of D727E polymorphism and GD (13).

To date, no compelling evidence exists to support a role of these three TSHR polymorphisms in GD pathogenesis. Given the frequency of these variants in general population, it is believed that they are common polymorphisms, not implicated in development of GD (20). The lack of consistency among completed studies could be the consequence of ethnic differences, selection bias, and population stratification.

In the last decade, the association of common genetic variants with complex diseases was significantly facilitated by the increased ability to measure genetic variability of hundreds of markers in large cohorts of individuals. The advent of genome-wide association technology applied to large case–control studies allowed identification of disease-associated variants and their contribution to disease susceptibility. Applying this technology to AITD resulted in identification of new disease-associated loci, including TSHR, and provided unique insights into their genetic contribution to disease pathology. Table 1 summarizes the main studies conducted over the years that established association of the TSHR gene with GD risk.

**Table 1:** **Association studies of thyroid-stimulating hormone receptor (TSHR) gene with Graves’ disease risk**.

Studies	Cases (*n*)	Population	Main polymorphisms found	Associated TSHR region
Cuddihy et al. (30)	91	Caucasian (USA)	rs2234919 (P52T)	Exon 1
Akamizu et al. (31)	186	Japanese	TSHR-AT	Intron 2
Chistiakov et al. (29)	78	Russian	rs1991517 (D727E)	Exon 10
Ho et al. (24)	164	Chinese, Malays, Indians	rs2239610	Intron 1
Hiratani et al. (32)	250	Japanese	rs2268475, rs3783938	Intron 7, intron 8
Dechairo et al. (5)	1,422	Caucasian (UK)	rs2268458	Intron 1
Burton et al. (33)	1,000	Caucasian (UK)	rs3783941	Intron 8
Yin et al. (34)	200	Caucasian (women only)	rs2268458	Intron 1
Brand et al. (6)	768	Caucasian (UK)	rs179247, rs12101255	Intron 1
Ploski et al. (35)	3,258	Caucasian (Poland, UK)	rs179247, rs12101255	Intron 1
Chu et al. (36)	5,530	Chinese	rs12101261	Intron 1
Colobran et al. (37)	137	Caucasian (Spanish)	rs179247	Intron 1
Liu et al. (38)	404	Chinese	rs12101255, rs179247	Intron 1
Inoue et al. (39)	112	Japanese	rs179247	Intron 1
Tomer et al. (18)	225	Caucasian (USA)	rs2284720	Intron 1
Liu et al. (40)	5,368	Chinese	rs12101261, rs179243	Intron 1
Bufalo et al. (41)	279	Brazilian	rs179247, rs12885526	Intron 1
Fujii et al. (42)	180	Japanese	rs4411444	Intron 1
Lombardi et al. (43)	333	Caucasian (Italy)	rs179247, rs3783948, rs12101255	Intron 1

In 2005, Dechairo et al. analyzed 40 SNPs mapping to a 600-kb sequence encompassing the TSHR gene in a Caucasian cohort of 1,056 AITD patients and 971 controls. They identified a haplotype associated with GD (OR = 1.7), but not with autoimmune hypothyroidism, and concluded that TSHR is a GD-specific susceptibility locus (5). Importantly, rs2268458, the SNP showing the strongest association (OR = 1.3) with GD mapped to TSHR intron 1; the association was confirmed in a large UK Caucasian cohort (5). The same year, an independent case–control study conducted in 400 patients with AITD and 238 controls of Japanese descent found several adjacent SNPs in TSHR intron 7 significantly associated with GD, but not with autoimmune hypothyroidism, suggesting that polymorphisms in the TSHR intron 7 could contribute to GD susceptibility (32). However, subsequent association studies conducted over several years in Caucasian populations could not replicate the association of TSHR intron 7 SNPs with GD (6, 35, 41).

In 2009, Brand et al. interrogated a panel of 98 SNPs spanning an 800 kb region of the TSHR gene in a cohort of 768 GD subjects and 768 controls (6). The SNPs showing the strongest association with GD, rs179247 (OR = 1.53), and rs12101255 (OR = 1.55) were located in TSHR intron 1 (6). The association of the TSHR intron 1 SNPs with GD was validated by further studies of several Caucasian (34, 35) and Brazilian (41) populations. In 2011, a large GWAS conducted by the China Consortium of the Genetics of Autoimmune Thyroid Disease in 1,536 individuals with GD and 1,516 controls confirmed TSHR as a primary susceptibility locus for GD by finding a robust association (OR = 1.35) of an intron 1 SNP (rs12101261) with the disease (36). Two years later, the same group conducted a fine-mapping study that established the association of two independent TSHR intron 1 susceptibility variants, rs1201261 and rs179243 in a large Chinese Han population (40). Noticeable, rs1201261 and rs179243 are in tight linkage disequilibrium (LD) with rs12101255 (*r*^2^ = 1.0) and rs2268458 (*r*^2^ = 0.91), respectively, which were found associated with GD in Caucasian populations of European descent (6).

Recently, three meta-analysis studies intended to refine the effects of rs179247 and rs12101255 SNPs on GD susceptibility concluded that there is a significant association between these SNPs in TSHR intron 1 and GD (44–46).

**Figure 1:**
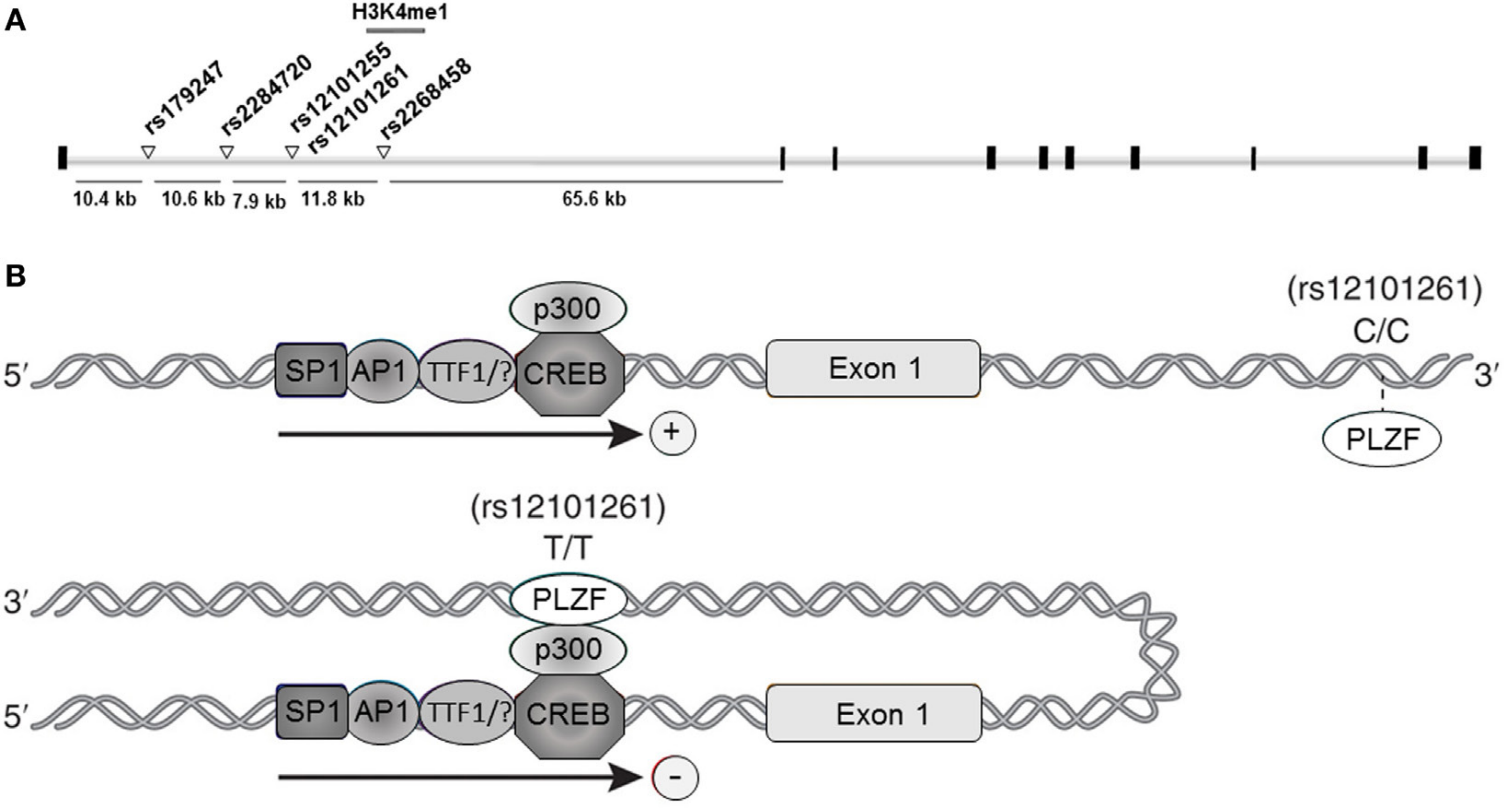
**Thyroid-stimulating hormone receptor (TSHR) intron 1 Graves’ disease (GD)-associated DNA variants**. **(A)** Schematic representation of the TSHR gene and the intron 1 GD-associated single nucleotide polymorphisms (SNPs). Black squares represent exons; white bars represent introns; white triangles in intron 1 represent the SNPs associated with GD susceptibility; gray bar overlapping the rs12101255 and rs12101261 represent the region characterized by H3K4me1 enrichment (47). **(B)** Proposed model for rs12101261 allele-dependent regulation of TSHR transcription. Upper panel: the presence of the disease-protective genotype (C/C) at the rs12101261 site prevents strong interactions with promyelocytic leukemia zinc finger protein (PLZF), allowing TSHR transcription; lower panel: the presence of the disease-associated genotype (T/T) facilitates binding of PLZF, triggering chromatin folding and interaction with TSHR promoter and transcriptional machinery.

Collectively, the association studies conducted during the last decade provided compelling evidence and established TSHR as a GD-specific susceptibility locus. Furthermore, fine-mapping studies pointed to a unique susceptibility region located in TSHR intron 1, where at least five GD-associated SNPs were mapped: rs179247, rs2284720, rs12101255, rs12101261, and rs2268458 (Figure 1A).

## Functional Relevance of TSHR Polymorphisms

The discovery of the TSHR GD-associated SNPs within a non-protein coding gene region raised questions about their potential effect on gene function, as well as their impact on disease pathology. Since DNA variants located in intronic or intergenic sites can impact different layers of gene regulation, identification of their functional role is often difficult. For example, by modifying the DNA sequence, non-coding SNPs can affect transcriptional factors’ (TFs) binding and thus modulate gene transcription; they can also impact RNA splicing and stability as well as posttranslation events (48). In addition, DNA variants can modulate, directly or indirectly, epigenetic marks such as DNA methylation, histone modifications, and microRNAs activity (48, 49). Recently, it has been shown that differential binding of TFs at sites harboring DNA variants triggers specific histone modifications, which can modify gene expression and determine the phenotype (50–52). In the case of disease-associated SNPs, such genetic–epigenetic interactions can increase the risk or even trigger disease in certain individuals.

To date, studies aimed to unveil the mechanistic role of TSHR intron 1 variants in gene function and thyroid autoimmunity pointed to two distinct mechanisms. The first proposed mechanism supports a role of the disease-associated SNPs in defective peripheral tolerance; the second mechanism supports the concept that the disease-associated intron 1 SNPs cause reduced intrathymic TSHR expression, leading to decreased central tolerance and increased risk of autoimmunity to TSHR.

Supportive of the first mechanism, Brand et al. proposed that the TSHR intron 1 GD-associated SNPs regulate mRNA spicing, resulting in increased levels of variants encoding a more auto-antigenic TSHR A-subunit (6). The authors measured the levels of full length TSHR (flTSHR) mRNA and of two TSHR truncated transcripts named ST4 and ST5 in thyroid tissues of 12 individuals and showed that the disease-risk alleles of 2 intron 1 SNPs (rs179247 and rs12101255) associate with increased ST4 and ST5 and with decreased flTSHR levels. They suggested that the truncated ST4 and ST5 variants could be translated into TSHR extracellular A-subunit, the main target of TSHR autoantibodies (6). However, the mechanisms by which the two SNPs interact with TSHR mRNA splicing were not addressed, and the authors did not exclude the possibility that other intron 1 SNPs in strong LD could also modulate TSHR transcription (53).

The second mechanism by which TSHR intron 1 variants could trigger thyroid autoimmunity through defective central tolerance was initially proposed by Pujol-Borrell group (37). By measuring TSHR mRNA levels in thymus and correlating them with the genotype of intron 1 SNPs, Colobran et al. found that individuals carrying the disease-protective genotype at the rs179247 site have higher levels of thymic TSHR mRNA than those with the disease-associated genotype (37). These findings, coupled with the fact that negative selection of autoreactive thymocytes is dose dependent (54), support the concept that, by modulating TSHR transcription, intron 1 disease-associated SNPs could modulate negative selection of TSHR-autoreactive T cells in the thymus. Thus, decreased TSHR thymic expression would facilitate the escape of TSHR-reactive T cells from central tolerance in genetically susceptible individuals, increasing the risk for AITD development. The same group showed that TSHR mRNA and protein are expressed in thymocytes from early stages of differentiation but are not detected in extra-thymic T cells. Moreover, thymic TSHR is functional, and TSAbs from GD patients can stimulate thymocytes through this receptor (55). Based on these findings, Pujol-Borrell group suggested that constant stimulation of thymic TSHR by TSAbs can be a potential mechanism explaining thymic hyperplasia, commonly observed in GD (55). Furthermore, the authors proposed that continuous TSAbs stimulation of thymocytes could lead to improved affinity and stimulating capability of TSHR cross-reactive low-affinity antibodies due to the interactions between egressing thymocytes and B-cells in the lymph nodes or the thyroid gland (55, 56). This would result in production of high-affinity TSAbs, the hallmark of GD pathogenesis.

Mechanistic insights into the contribution of TSHR intron 1 SNPs to AITD susceptibility through defective central tolerance were recently revealed by studies from Tomer group (47). Their work originated from the premise that the disease-associated variants can specifically interact, through epigenetic modifications, with environmental factors to trigger disease susceptibility. To reveal the functionality of the GD-associated SNPs, Stefan et al. (47) analyzed genome-wide modifications of histone 3 lysine 4 (K4)-monomethylated (H3K4me1), a chromatin mark often associated with distal enhancer elements (57), induced by interferon alpha (IFNα), a key cytokine secreted during viral infections, previously shown to trigger thyroid autoimmunity (58).

This approach led to identification of an open chromatin region marked by IFNα-induced H3K4me1 enrichment overlapping two adjacent TSHR intron 1 GD-associated SNPs: rs12101255 and rs12101261 (47) (Figure 1A). Functional studies revealed that the region overlapping the rs12101261 site harbors a regulatory element that functions through binding of the transcriptional repressor, promyelocytic leukemia zinc finger protein (PLZF). PLZF binding was shown to be restricted at the disease-associated variant of the rs12101261 site and was correlated with lower thymic TSHR mRNA levels in individuals carrying the disease-predisposing genotype, as compared with individuals carrying the disease-protective genotype. The authors proposed that loss of proper genetic–epigenetic interactions due to microenvironmental influences, such as sustained IFNα production during viral infections, would affect the regulation of TSHR susceptible variants resulting in impaired gene expression. In thymus, the lower TSHR expression triggered by the susceptible genotype would likely facilitate escape from central tolerance and increases the risk of autoimmunity to TSHR.

However, the underlying mechanisms by which the *cis*-regulatory element at the rs12101261 site modulates TSHR transcription have still to be experimentally addressed. A possible regulatory model involves long-range chromatin interactions determined by the rs12101261 genotype. In such model, the presence of the disease-protective genotype (C/C) would cause a weak interaction of PLZF with chromatin at the rs12101261 site, resulting in active TSHR transcription (Figure 1B, upper panel). The presence of the disease-associated genotype (T/T) would enable strong PLZF binding at the *cis*-element, triggering formation of chromatin loops and direct interactions between PLZF and TSHR promoter, resulting in inhibition of transcription (Figure 1B, lower panel). Thus, allele-dependent differences in chromatin folding would trigger allele-dependent differences in gene expression. These chromatin interactions, still to be experimentally addressed, are likely cell specific, and different factors (e.g., TFs) and SNPs may control TSHR expression in different tissues.

## Concluding Remarks

Studies in the last 15 years established a robust association of TSHR gene with GD, and the disease-associated locus was recently fine-mapped within 40 kb region in intron 1. Moreover, it has become clear that whether TSHR SNPs interfere with gene expression in thymus leading to the escape of TSHR-reactive T cells from central tolerance or defects in peripheral tolerance are involved, these variants are unlikely to act alone, and interactions with epigenetic and environmental factors as well as combinatorial effects should be considered.

Although important advances have been made in our understanding of the role of TSHR polymorphisms in AITD, questions still persist. What are the mechanisms by which TSHR polymorphisms predispose to disease? Which are the environmental factors that unequivocally contribute to disease development and how they interact with susceptible variants? Can genetic variants be translated into markers predicting disease development? Does susceptibility of epigenetic markers to environmental triggers have a role in the functionality of the disease-associated variants? To answer these questions, more work and close collaborations of molecular biologists and clinical scientists as well as more integrated approaches are needed. It is hoped that such knowledge would open the road toward the development of new, targeted, and preventive therapies based on the individual’s particular susceptibility.

## Author Contributions

All authors listed have made substantial, direct, and intellectual contribution to the work and approved it for publication.

## Conflict of Interest Statement

The authors declare that the research was conducted in the absence of any commercial or financial relationships that could be construed as a potential conflict of interest.
